# Assessment of Behavioral Risk Factors in Chronic Obstructive Airway Diseases of the Lung Associated with Metabolic Syndrome

**DOI:** 10.3390/jcm13041037

**Published:** 2024-02-11

**Authors:** Andreea Mihai, Magdalena Mititelu, Marius Matei, Elena Carmen Lupu, Liliana Streba, Ionela Mihaela Vladu, Maria Livia Iovănescu, Ramona Cioboată, Cristina Călărașu, Ștefan Sebastian Busnatu, Costin Teodor Streba

**Affiliations:** 1 Department of Pulmonology, University of Medicine and Pharmacy of Craiova, 200349 Craiova, Romania; andreea.mihai@umfcv.ro; 2 Faculty of Pharmacy, “Carol Davila” University of Medicine and Pharmacy, 020956 Bucharest, Romania; ramona.cioboata@umfcv.ro (R.C.); cristina.calarasu@umfcv.ro (C.C.); costin.streba@umfcv.ro (C.T.S.); 3 Department of Histology, University of Medicine and Pharmacy of Craiova, 200349 Craiova, Romania; 4 Faculty of Pharmacy, “Ovidius” University of Constanta, 900470 Constanta, Romania; clupu@univ-ovidius.ro; 5 Department of Oncology, University of Medicine and Pharmacy of Craiova, 200349 Craiova, Romania; liliana.streba@umfcv.ro; 6 Department of Diabetes, Nutrition and Metabolic Diseases, University of Medicine and Pharmacy of Craiova, 200349 Craiova, Romania; ionela.vladu@umfcv.ro; 7 Department of Cardiology, University of Medicine and Pharmacy of Craiova, 200349 Craiova, Romania; maria.iovanescu@umfcv.ro; 8 Department of Cardio-Thoracic Pathology, Faculty of Medicine, “Carol Davila” University of Medicine and Pharmacy, 050474 Bucharest, Romania; stefan.busnatu@umfcd.ro

**Keywords:** eating habits, junk food, pulmonary obstructive ventilatory dysfunction, metabolic disorders, lifestyle

## Abstract

Background: Diet and lifestyle play important roles in preventing and improving chronic diseases, and evaluating behavioral risk factors in these pathologies allows for efficient management. Methods: A clinical study by screening biochemical parameters and pulmonary function was carried out to evaluate behavioral risk factors in obstructive pulmonary disease associated with metabolic syndrome. Results: Of the total of 70 patients included in the clinical study, 46 were men and 24 were women (χ^2^ = 3.9, *p* = 0.168). Forty-eight patients presented at least three met risk criteria associated with the metabolic syndrome (19 women and 29 men). Regarding the assessment of lung function, only 7 of the patients presented normal spirometry values (χ^2^ = 75.28, *p* < 0.001), and the other 63 patients presented with ventilatory dysfunction; most (over 80%) declared that they were smokers or had smoked in the past (χ^2^ = 5.185, *p* = 0.075). In terms of body weight, 45 of the patients are overweight or obese, most of them declaring that they do not consume enough vegetable products, they consume large amounts of foods of animal origin (meat, milk, eggs) but also super processed foods (food products type of junk food), do not hydrate properly, and are predominantly sedentary people (54 of the patients do no physical activity at all; χ^2^ = 2.12, *p* = 0.713). Conclusion: From the statistical processing of the data, it is noted that insufficient hydration, low consumption of vegetables, increased consumption of hyper-caloric food products rich in additives, sedentary lifestyle, and smoking are the main disruptive behavioral factors that worsen the health status in lung disease associated with the metabolic syndrome. An important conclusion emerging from the study is that the imbalances that aggravate obstructive lung diseases are generated by unhealthy food and an unbalanced lifestyle.

## 1. Introduction

Despite their distinct pathophysiology, Chronic Obstructive Pulmonary Disease (COPD) and Asthma exert a profound impact on respiratory health. Characterized by chronic inflammation, airway obstruction, and debilitating symptomatology, they not only pose a physical and emotional burden on millions of people, with a direct impact on lowering life quality, but they also represent a substantial economic challenge for healthcare systems, thereby affecting society worldwide [1].

Considering its multiple environmental and genetic risk factors, one still stands out, holding the leading position for COPD: tobacco smoking. With recent data suggesting that over 200 million individuals are living with this diagnosis, the major concern is that this staggering number is expected to rise. Globally, this condition has been assigned third place among the most prevalent causes of death, intensifying the emotional distress of patients facing this condition [2,3,4].

Although over the years the primary concern regarding COPD was focused on the high rates of morbidity observed among the elderly, current guidelines are also directing our attention towards the concept of ‘young COPD’, emphasizing the importance and the benefits of early disease diagnosis for patients within the age range of 20 to 50 years [4,5].

Another public health challenge, Asthma exerts substantial influence across all age groups, with a significant impact during childhood. Viral or bacterial respiratory infections, environmental allergens, and certain air pollutants represent a small part of the triggers involved in exacerbations [6,7].

Both asthma and COPD are responsible for high mortality rates, particularly in low- and middle-income areas. To improve disease control and overall survival, it is mandatory to adopt a comprehensive approach to the cases and consider and treat comorbidities in direct correlation with associated risk factors [4,8].

Multimorbidity, the coexistence of multiple chronic conditions in an individual, is a widespread issue significantly impacting patients with obstructive lung diseases. Therefore, clinicians encounter cases with more severe symptoms, a higher risk of drug interactions, and an increased probability of undesired therapeutical outcomes [9,10].

Recent treatment guidelines emphasize the importance of addressing and managing comorbidities in both asthma and COPD. While the specific conditions may vary, cardiovascular diseases, diabetes, metabolic syndrome, gastroesophageal reflux, and depression are among the most frequently encountered [4,11].

Assessing all these, metabolic syndrome (MetS) notably sets itself apart due to its alarmingly increasing incidence and complex pathogenesis. With rapid urbanization, changing lifestyles, and dietary habits, it is estimated that this multifaceted syndrome has reached a global prevalence of about 20 to 25% of the adult population [12].

MetS represents a cluster of conditions, including high blood pressure, central obesity, insulin resistance, and atherogenic dyslipidemia, that can occur together and thus significantly increase the patient’s risk of cardiovascular disease by approximately twofold and amplify the probability of developing type 2 diabetes by fivefold [13].

The link between MetS and obstructive lung diseases has gained attention in recent medical research. While some studies emphasized the idea that obesity, a key characteristic of MetS, represents a risk factor for both the onset and the severity of asthma, others delve deeper into the issue, suggesting that changes in hormones originating from adipose tissue, such as leptin and adiponectin, influenced by obesity, might negatively impact the asthmatic profile. Furthermore, hyperinsulinemia in correlation with insulin resistance, another core feature of MetS, might impact the pathogenesis of this condition considering its possible effects of growing airway smooth muscle mass and enhancing contractile function, as observed in the experimental analysis [14,15,16].

In addition, it was found that MetS could influence the progression and outcomes of COPD, highlighting the possibility of shared inflammatory pathways [17,18,19].

Considering all this invaluable data, there is still a recurring emphasis on the imperative need for more comprehensive approaches to facilitate the management of patients dealing with these conditions.

This study aims to determine and evaluate anthropometric and biological parameters utilized to diagnose MetS among patients admitted for obstructive pulmonary diseases. Additionally, a comprehensive analysis of potentially modifiable lifestyle factors was conducted to assess their associated risk regarding the occurrence of metabolic syndrome among patients with COPD or Asthma.

## 2. Materials and Methods

### 2.1. Study Design

To establish an appropriate management strategy for patients who present both obstructive bronchial pathology and metabolic syndrome, we conducted a comprehensive cross-sectional, non-interventional clinical study involving patients with chronic obstructive pathology selected from the pneumology clinics of two hospitals.

Hence, we included a total of 70 consecutive patients hospitalized in the pneumology departments of Leamna Clinical Pneumophthisiology Hospital and, respectively, from The Clinical Hospital of Infectious Diseases and Pneumophthisiology “Victor Babeș” from Craiova, between 1 March 2023, and 3 July 2023, in the Oltenia region. The number of participants in the study was calculated using G*Power version 3.1.9.4 [20]. Based on a previous study which indicates a prevalence of metabolic syndrome among the Romanian population of 38.5% [21] and taking into account the Romanian population reported at the last census [22], 66 participants were required, with an effect size of 0.38 at a significance level of 0.05, and a power of 0.90.

Subject participation in the study was voluntary, following the presentation and signing of the informed consent. The present research adhered to the ethical principles outlined in the updated Declaration of Helsinki and received approval from the Ethics Committee of the University of Medicine and Pharmacy in Craiova (No.143/04.07.2023). The patients participated in the study after giving their informed consent voluntarily, without any political, social, or religious discrimination, and in compliance with data protection laws.

The inclusion criteria aimed to enroll patients aged 18 and above, with informed consent for study inclusion, hospitalized for the diagnosis of Chronic Obstructive Pulmonary Disease (COPD) or asthma. Patients were assessed based on specific criteria, with details regarding pulmonary function, and were required to be out of infectious exacerbations and suitable for study participation. The participants enrolled in the study had previously been hospitalized for routine clinical assessments. We included subjects during stable clinical conditions to accurately assess their clinical, paraclinical, and respiratory functional status, avoiding the potential negative impact of infectious exacerbations on lung volumes.

Subjects were allocated into two distinct cohorts based on the presence or absence of meeting the criteria for metabolic syndrome as follows:

Cohort 1: Patients with COPD or asthma who had associated metabolic syndrome.

Cohort 2: Patients with COPD or asthma who did not meet the conditions for the diagnosis of metabolic syndrome.

The updated version of the guideline established by the National Cholesterol Education Program—Adult Treatment Panel III (NCEP-ATP III) was used to define the presence of metabolic syndrome. All measurements were conducted following the specified recommendations. According to this definition, metabolic syndrome is defined by the presence of at least three of the following criteria [23]:

1. Abdominal obesity defined by waist circumference:
  ≥102 cm for men;
  ≥88 cm for women.
2. Hypertriglyceridemia defined by triglyceride levels:
  ≥150 mg/dL or hypotriglyceridemic medication.
3. Low HDL cholesterol:
  <40 mg/dL for men or medication for reduced HDL cholesterol;
  <50 mg/dL for women or medication for reduced HDL cholesterol.
4. Increased blood pressure (BP):
  - systolic BP (SBP) ≥130 mmHg and/or diastolic BP (DBP) ≥85 mmHg or antihypertensive medication.
5. Hyperglycemia:
  - fasting glucose ≥100 mg/dL or antidiabetic medication.

Exclusion criteria for both cohorts of patients were represented by situations in which the progressive stages of associated conditions did not permit the proposed evaluation. The presence of additional comorbidities that would significantly impact the overall health status of the patients was considered, leading to the exclusion from the study of any patient with severely compromised general health. Considering that all patients underwent respiratory functional assessment, subjects with symptoms or pathologies overlapping absolute or relative contraindications of spirometry were not included. The study prioritized the well-being of the participants, and we made a deliberate decision to exclude individuals with significantly compromised health status resulting from concurrent comorbidities. This exclusion aimed to ensure the robustness of our designated study cohort’s clinical, biological, and respiratory functional data. The rationale behind this decision was rooted in the anticipation that patients with markedly altered clinical statuses might present biological markers strongly influenced by the severity of other severe health conditions. Recognizing spirometry as a demanding test that necessitates respiratory effort, we acknowledged the potential of aggravating underlying conditions or even encountering contraindications for the test in specific cases. Additionally, clinical questionnaires thoroughly assessed all participants’ lifestyle and dietary habits. Thus, inclusion criteria emphasized patient cooperation and the absence of severe neurological impairment. Moreover, anthropometric measurements required a clinical state facilitating unassisted orthostasis—a condition unattainable for those with severely altered statuses.

During the enrollment, participants underwent a comprehensive medical history review, particularly emphasizing cardiovascular, metabolic, and pulmonary elements of interest. Subsequently, anthropometric measurements, including height and weight, were taken. The same personnel consistently conducted these measurements using standardized medical equipment: OMRON Body Composition Monitor BF511 HBF-511T-E/HBF-511B-E (OMRON HEALTHCARE Company; Kyoto, Japan). Blood pressure was determined using a calibrated automatic sphygmomanometer (OMRON X3 Comfort HEM-7155-EO; Omron Company; Kyoto, Japan). The evaluations were conducted following the guidelines provided by the American Heart Association (AHA).

Within the research, venous blood samples of 6 milliliters per patient were collected to assess various essential biological parameters needed for evaluating elements regarding metabolic syndrome. These parameters included fasting blood glucose, triglycerides (TGL), high-density lipoprotein (HDL) cholesterol, and low-density lipoprotein (LDL) cholesterol. The analysis was performed using the BioSystems A15 automatic analyzer (BioSystems Company, Barcelona, Spain).

All enrolled patients underwent respiratory functional assessment through spirometry using Spirolab IV (Medical International Research Company; Roma, Italy). We recorded both the absolute values and the percentage of predicted values for forced expiratory volume in one second (FEV1) and forced vital capacity (FVC) and calculated the FEV1/FVC ratio for each patient.

All patients were asked to respond to three standardized questionnaires (used in evaluation studies of behavioral risk actors and studies published in specialized journals). The first two surveys aimed to evaluate various aspects of lifestyle and eating habits, specifically focusing on the consumption of non-alcoholic beverages and unhealthy food products [24,25,26]. To assess the behavioral risk factors in correlation with the patient’s biochemical parameters, they measured the frequency of consumption of junk food, alcoholic beverages, sweetened beverages, sweets, vegetables, fruits, dairy products, pasta, fish, meat, and eggs, along with physical activity and smoking. The third one included queries designed to assess the impact and severity of symptoms related to pulmonary obstructive diseases, employing the COPD Assessment Test (CAT) and the Modified Medical Research Council (MMrc) Dyspnea Scale [27]. The obstructive ventilatory dysfunction (DVO), the restrictive ventilatory dysfunction (DVR), and the mixed ventilatory dysfunction (DVM) were evaluated.

The Body Mass Index (BMI) was calculated using the formula [body mass (kg)/height (m^2^)], involving the processing of the anthropometric data [28,29].

The chosen approach for survey registration was an online platform using Google Forms. To ensure the correct recording of responses in accordance with the patient’s order number within the study, a physician was trained to read each question to the included individuals, enumerate response options, repeat them if necessary, and finally, mark the corresponding checkbox with every single answer received.

The initial assessment of patients involved studying them in correlation to a set of general characteristics. Therefore, for each patient group, the distribution of cases based on gender and age distribution was specified.

### 2.2. Statistical Analysis

The background attributes of the subjects included in the study were outlined using descriptive statistics. For categorical variables, absolute frequencies (n) and relative frequencies (%) were employed for presentation. To discern conceivable relationships between behavioral risk factors (diet, physical activity, tobacco use) and anthropometric data (gender and BMI), a simple correspondence analysis was employed.

The Kolmogorov–Smirnov test was used to assess the normality of the distribution of variables. Spearman’s correlation test was used for variables that did not present normal distribution. In the comparison between two independent groups for variables with n < 30 or without normal distribution, the Mann–Whitney’s test was performed.

To examine the hypothesis of dependence between categorical variables, a Chi-square test was conducted. A receiver operating characteristics (ROC) curve analysis, quantified by the area under the ROC curve (AUC), was used to assess the values of the indicators (age, fasting blood glucose, abdominal circumference, BMI, TGL, and FEV) for predicting MetS. Youden’s index (sensitivity + specificity − 1) was used to determine the optimal cut-off point of each index.

The statistical outcomes regarding the consumption frequency of soft drinks are derived from two-sided tests. During the conclusive phase of the analysis, we investigated the relationship between the variable “Quantity of junk food” and independent variables, including gender and BMI groups. The statistical analysis employed XLSTAT (version 2020, Addinsoft, New York, NY, USA) for Correspondence and the Statistical Package for the Social Sciences, version 23 (SPSS Inc., Chicago, IL, USA). A significance threshold of *p* < 0.05 was considered for statistical significance [30,31,32].

To ensure the homogeneity and integrity of the clinical database used in the statistical analysis, patients who had contraindications to some clinical evaluations due to severe health conditions were not included in the study. Thus, patients with severe metabolic syndrome and health-altered conditions were excluded from the evaluation group.

## 3. Results

The average age of the patients in the study was 62.42 ± 10.93. Regarding the abdominal circumference in the persons included in the studied group, an average value of 103.38 ± 15.89 cm was observed. The average abdominal circumference in men was 105.39 ± 15.56, and in women, 99.54 ± 16.12. The statistical analysis showed that out of the total of 70 people included in the study group, 48 of them (68.57%) had at least three metabolic syndrome factors.

At the time of lung function evaluation by spirometry, 63 patients presented ventilatory dysfunction (χ^2^ = 75.28, *p* < 0.001). The majority of the patients (80.95%) declared that they were smokers or ex-smokers (χ^2^ = 5.185, *p* = 0.075). Most of the patients (Table 1), according to the processing of the anthropometric data, are overweight (22 patients) or obese (23 patients), and in the group of patients with detected metabolic syndrome, patients were predominantly obese and overweight; 23 patients were normal weight from the study group and there were 2 underweight patients (χ^2^= 16.59, *p* = 0.006). Most of the patients, i.e., 54 of them, declared that they do not engage in any physical activity at all, and 75% of them presented metabolic syndrome according to the evaluations (χ^2^ = 2.12, *p* = 0.713). Among the patients with metabolic syndrome, 45.8% declared that they work in dangerous conditions (χ^2^ = 6.266, *p* = 0.394), and 89.6% consider that they eat a balanced diet (χ^2^ = 4.76, *p* = 0.092) without excesses, which means that either they do not realize that they are eating excessively or the sedentary lifestyle is the leading cause of excess weight and the consumption of hyper-caloric junk food.

A negative correlation between FEV1 and BMI was found (r = 0.310, *p* = 0.009). FEV1 correlates negatively with SBP (r = −0.234, *p* = 0.049). Also, in addition to BMI and age, it is a factor that influences the occurrence of metabolic syndrome (r = 0.304, *p* = 0.017).

The data presented in Table 2 indicate a negative correlation between the consumption of packs of cigarettes per year and lung function: patients with an increased tendency to smoke cigarettes show a greater reduction in FEV1. However, both age and the severity of the metabolic syndrome might accentuate the degradation of lung function. Thus, in the case of patients with a higher number of metabolic syndrome factors, tobacco consumption might affect lung function more compared to those with fewer factors of metabolic syndrome, even if, in some situations, tobacco consumption is higher among those in the latter category.

### 3.1. Metabolic Syndrome Risk Assessment

**Figure 1:**
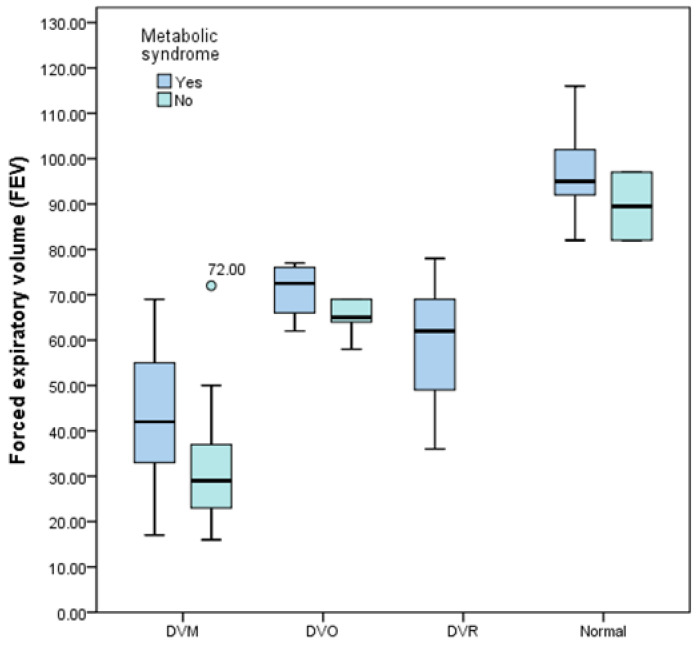
Box-and-whisker graph of the risk of metabolic syndrome associated with the degree of severity of obstructive pulmonary disease.

The correlation between the risk of metabolic syndrome and the results of the evaluation by spirometry, as well as the FEV, are shown in Figure 1:

- For risk score 0: a total of two patients, a female patient aged 48 and a male patient aged 63. Both patients are of normal weight.
- Spirometry results for SCOR 0 (0 risk criteria fulfilled): moderate DVO for both patients.
- For risk score 1: a total number of five patients, all male, average age 57 years, four normal weight patients, one slightly overweight.
- SCOR 1 spirometry results (1 risk criterion fulfilled): DVO—moderate one patient, moderate-severe DVM one patient, and very severe DVM three patients (60%).
- For risk score 2: a total number of 15 patients, 4 women and 11 men, average age of 57.26 years, 1 underweight patient, 8 normal weight patients, 6 overweight or obese patients.
- Spirometry results for SCOR 2 (2 risk criteria met): normal two patients, DVO—moderate one patient, moderate-severe DVO one patient, mild DVM one patient, severe DVM three patients, and very severe DVM three patients; DVM type (mixed ventilatory dysfunction) predominates in 11 patients, i.e., 73.33%.
- For risk score 3: a total number of 12 patients, 6 women and 6 men, average age of 63.25 years, 1 underweight patient, 7 normal weight patients, 4 obese patients.
- Spirometry results for SCOR 3 (3 risk criteria fulfilled): normal two patients, DVO—moderate one patient, moderate DVM one patient, moderate-severe DVM one patient, severe DVM three patients, very severe DVM three patients, mild DVR one patient; DVM type (mixed ventilatory dysfunction) predominates in eight patients, i.e., 66.66%.
- For risk score 4: a total number of 12 patients, 3 women and 9 men, average age of 63.25 years, 2 normal weight patients, 6 overweight patients and 4 obese patients.
- Spirometry results for SCOR 4 (4 risk criteria fulfilled): normal one patient, DVO—mild one patient, moderate-severe DVM two patients, severe DVM three patients, very severe DVM three patients, moderate DVR one patient, severe DVR one patient; DVM type (mixed ventilatory dysfunction) predominates in eight patients, i.e., 66.66%.
- For risk score 5: a total number of 24 patients, 10 women and 14 men, average age of 66.54 years, 11 overweight patients and 13 obese patients.
- Spirometry results for SCOR 5 (5 risk criteria fulfilled): normal two patients, DVO—mild two patients, DVM moderate two patients, DVM moderate-severe three patients, DVM severe five patients, DVM very severe three patients, DVR mild one patient, DVR moderate three patients, DVR moderate-severe two patients, DVR severe one patient; DVM type (mixed ventilatory dysfunction) predominates in 13 patients, i.e., 54.16%.

### 3.2. Analysis of Behavioral Risk Factors

Table 3 shows the correlations between the different parameters involved in the onset of the metabolic syndrome. A positive correlation is observed between metabolic syndrome, pulmonary respiratory dysfunction, blood pressure, and the serum level of triglycerides, and a negative correlation between the serum level of HDL-cholesterol, metabolic syndrome, pulmonary respiratory dysfunction, and blood pressure.

**Figure 2:**
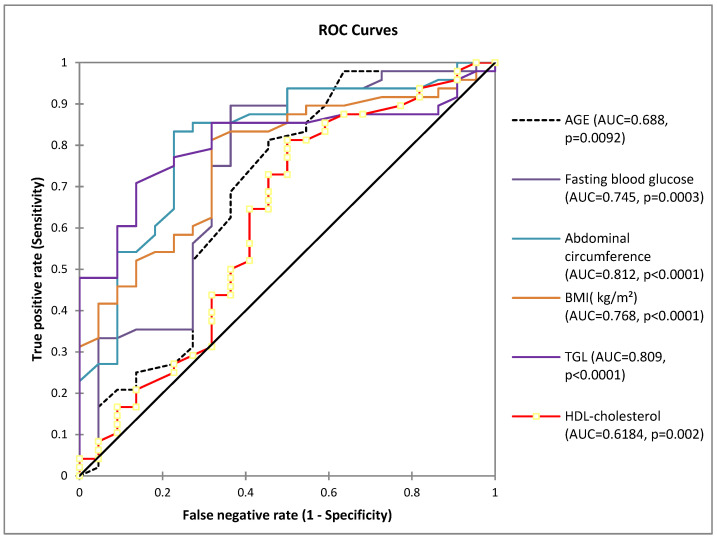
Receiver operating characteristic curves (ROC) for the parameters involved in the risk of metabolic syndrome (AUC = area under the curve). The diagonal represents the no-effect line (AUC = 0.50).

The ROC curve highlighted that age, with a probability of 68.8%, has an important role in the occurrence of metabolic syndrome (Figure 2). The optimal cut-off value of age to detect the metabolic syndrome with maximum sensitivity and specificity was 57 years old, with a positive predictive value (PPV) of 79.5% and a negative predictive value (NPV) of 57.1%. For fasting blood glucose, the optimal cut-off value was 98 (mg/dL), positive predictive value (PPV) 84.3%, and negative predictive value (NPV) 73.6%. For abdominal circumference, the optimal cut-off value was 89 cm, positive predictive value (PPV) 88.8%, and negative predictive value (NPV) 68.0%. For TGL, cut-off value was 105 mg/dL, the positive predictive value (PPV) was 91.8%, and the negative predictive value (NPV) was 57.5%.

**Figure 3:**
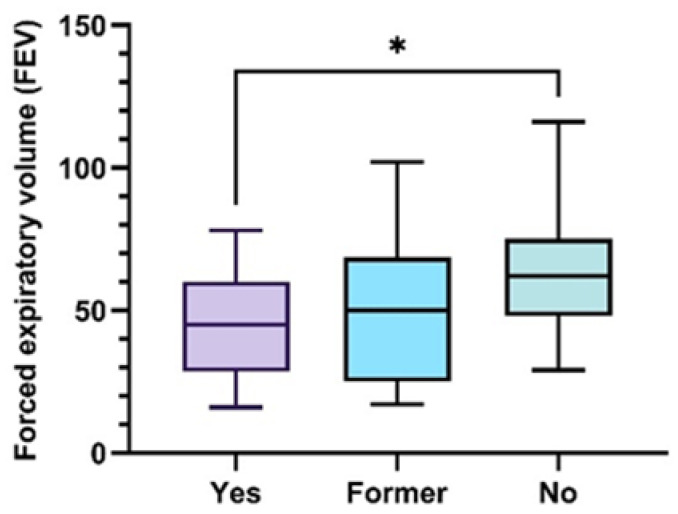
Box-and-whisker graph of FEV as a function of tobacco consumption 25th and 75th percentiles with median in between (ANOVA, with post hoc Tukey test), * *p* ˂ 0.05.

A strong negative correlation was found between the habit of smoking (smokers or ex-smokers = Former) and the FEV1 value (F = 3.494, *p* = 0.040), smoking being an important risk factor for obstructive pulmonary disease (Figure 3).

**Figure 4:**
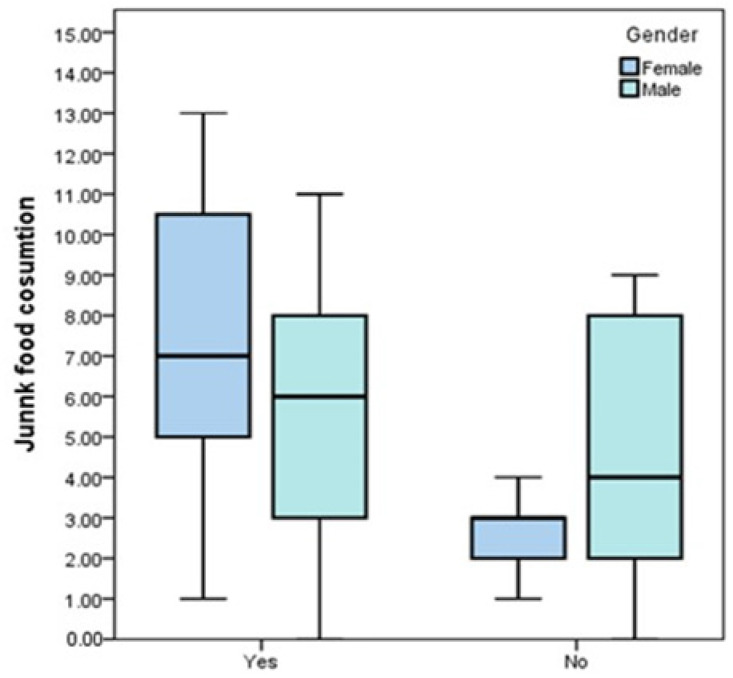
Box-and-whisker graph of FEV according to the average number of various junk food type products consumed, gender, and the presence of metabolic syndrome.

The evaluation of the consumption of junk food consumption (sweets, fast food products, fried potatoes, pastries, candies, chewing gum, sweetened carbonated and non-carbonated drinks, and coffee) was carried out by quantifying the number of various products consumed, but also by quantification of consumption frequency (Figure 4). There is a greater tendency to consume hypercaloric processed foods among patients with metabolic syndrome.

**Figure 5:**
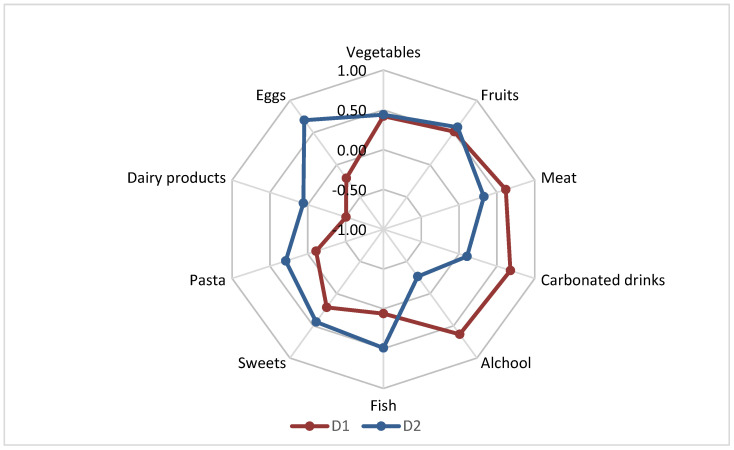
Factor loadings after varimax rotation on identified dietary patterns. % variance explained: Metabolic syndrome-YES D1: 19.30%; Metabolic syndrome-NO: 16.88. Total variance explained: 36.18%.

Exploratory factor analysis was performed to identify underlying dietary patterns using the average portions consumed. Bartlett’s test of sphericity (*p* = 0.010) supported the appropriateness of factor analysis. D1 (Metabolic syndrome-YES) had high positive loadings on alcohol and carbonated drinks, and negative loading on dairy products. D2 (Metabolic syndrome-NO) was characterized by high positive loadings on fish, eggs, and pasta (Figure 5).

Correspondence analysis (CA) was conducted for variables of BMI, gender, spirometry, and the number of met metabolic syndrome conditions.

The bi-plot indicates that 83.78% of the variability observed can be attributed to the two main components for F1 (59.30%) and F2 (24.48%).

**Figure 6:**
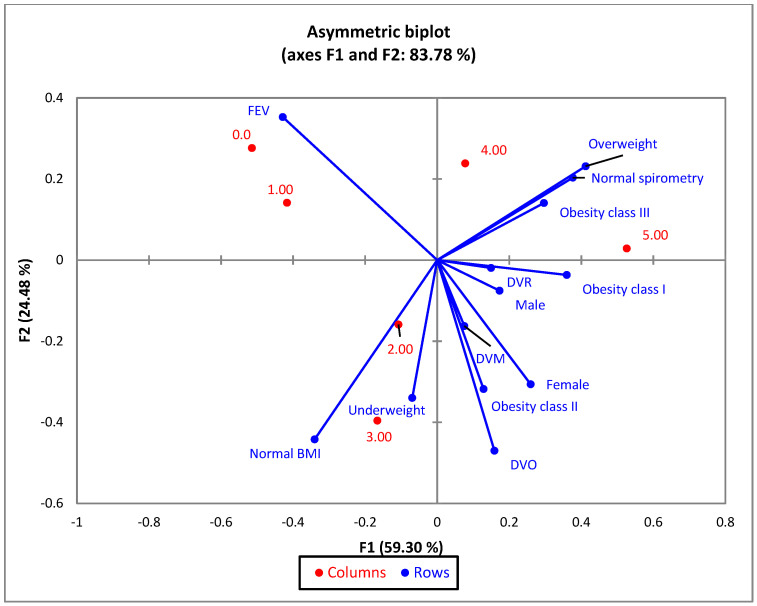
The first two dimensions of the correspondence analysis (CA) symmetric plot using BMI groups and the results of the spirometry tests as rows, and all 5 metabolic syndrome risk criteria as columns.

Those presenting 5 and 4 criteria are mostly male, generally having a body mass index > 25.00. The higher the number of fulfilled conditions, the lower the mean FEV1 values (Figure 6). As patients meet a greater number of metabolic syndrome conditions, there is a tendency for an increase in BMI and a decrease in FEV1, but also an increase in pulmonary dysfunctions (DVO, DVR, DVM). Patients with a low metabolic syndrome score (0 and 1) have increased FEV1 values, and those with a high metabolic syndrome score (4 and 5) tend to have a more pronounced pulmonary function impairment.

**Figure 7:**
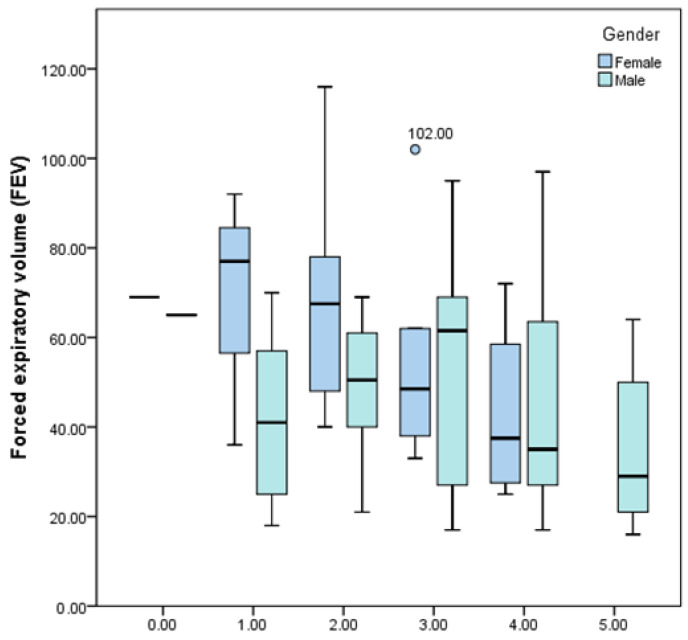
Box-and-whisker graph of FEV1 depending on the number of met metabolic syndrome risk criteria and gender.

The forced expiratory volume in one second (FEV1) decreases as more metabolic syndrome risk criteria are met; the decrease is more pronounced in female patients (Figure 7).

## 4. Discussion

Among the 70 patients included in the clinical study, 46 are men (65.71%) and 24 are women (34.29%). According to the analysis of metabolic syndrome association criteria, 48 patients meet at least 3 risk criteria, 19 women and 29 men, so a higher prevalence of metabolic syndrome is seen among female patients (79.16%) compared to male patients, where the percentage is 63.04% (Table 1). Men tend to consume a more diverse range of junk food products compared to women; as regards the average number of junk food products consumed, patients with metabolic syndrome consume a narrower range of products compared to those without metabolic syndrome (Figure 4). Regarding the frequency of consumption, men and especially people with metabolic syndrome tend to more frequently consume junk food and ultra-processed and high-calorie products.

People with metabolic syndrome are used to consuming small amounts of vegetable products (maximum 1 or 2 portions of 100 g per day or every 2-3 days) and even dairy products, over 50% consume water up to one liter per day, and they very rarely consume fish; instead, they consume large amounts of meat, carbonated drinks, and alcohol (Figure 5). People without metabolic syndrome consume larger quantities of vegetable products and hydrate better.

According to the statistical correlations in Figure 6, a worsening of obstructive pulmonary disease (accentuation of ventilatory dysfunction) is observed as the severity of the metabolic syndrome increases, and men present a higher risk compared to the women included in the study. There are studies in specialized literature that indicate that obstructive pulmonary disease associated with metabolic syndrome, especially in conditions of increased insulin resistance and obesity, elevated the associated risk of morbidity and mortality, especially due to cardiovascular complications [33,34,35]. A meta-analysis encompassing eleven studies concluded that, excluding the effects of hypolipidemic interventions on the serum lipid profile, patients with COPD had higher triglyceride levels than subjects without this condition [36]. Increased levels of serum triglycerides were also linked to the occurrence of asthma in individuals with obesity, suggesting that elevated triglycerides could represent a potential characteristic contributing to the onset of asthma [37,38].

Smoking, genetic factors, physical inactivity, and unhealthy diet are also incriminated as aggravating factors [39,40].

Pulmonary function is affected by the severity of the associated metabolic syndrome, and also by behavioral risk factors, as shown in this clinical study. Some studies indicate an alteration of the spirometry parameters with the severity of the metabolic syndrome, especially when the patients also have significant cardiovascular diseases [41,42,43,44]. Several epidemiological investigations revealed an association between low levels of serum high-density lipoprotein cholesterol (HDL-C) and the occurrence of wheezing [45,46]. Multiple studies observed that abdominal obesity had a notable negative impact on respiratory functions [47,48]. Additional findings show that an increase in abdominal obesity markedly lowers the asthma control score [49,50]. Specialists draw attention to the need to balance patients with comorbidities through diet and adequate rest [47,48]. Similar negative correlations have been observed in patients with COPD. Several studies indicated that abdominal obesity is associated with increased comorbidities and poorer therapeutical outcomes [51,52]. Along with the hyper-caloric diet rich in saturated fats, the specialized literature indicates two more important behavioral risk factors involved in the deterioration of lung function: sedentary lifestyle and smoking [53,54,55,56,57].

The limitations of the study are determined by the advanced age of the patients, the lower presence of female patients, and the uneven distribution of the patients on different degrees of risk in relation to the metabolic syndrome. Also, accurate quantification of cigarette packs smoked by patients was difficult to achieve. Through extensive discussions with the participants, especially active smokers, we observed difficulties in accurately quantifying the number of cigarettes smoked daily and providing precise data on the years of smoking. Many participants recounted various attempts of cessation, undergoing multiple stages in their smoking habits throughout the course of the disease. Factors such as worsening clinical status during exacerbations, financial constraints, motivation derived from participation in educational programs, or fear of developing bronchopulmonary neoplasms following the diagnosis of similar cases in acquaintances contribute to periods of tobacco cessation. On the other hand, many patients reported that during certain periods of emotional stress, or conversely, during leisure and relaxation, when they had the opportunity to spend more time with friends, there was a discernible escalation in their smoking habits. Another notable limitation of this study is excluding patients with severely compromised clinical status. This decision was made to preserve the integrity of the evaluation process at each stage of the study. The practical aspects of patient participation, including the need for ortho-stasis during anthropometric measurements, patient cooperation in answering lifestyle assessment questionnaires, and the demanding nature of spirometry with multiple contraindications, influenced this exclusion. The study’s cross-sectional design inherently limits our ability to infer causality, as it disallows establishing temporal precedence and determining a clear cause-and-effect relationship between metabolic syndrome and chronic obstructive airway diseases. While associations are discerned, the direction of influence remains uncertain. Future longitudinal investigations are needed to unravel the nuanced dynamics between the pathological entities mentioned before offering a more comprehensive understanding of their interrelationship. The observations can only be indicative and important to formulating a series of recommendations related to nutrition and lifestyle in order to improve the patient’s quality of life.

The importance of the study lies in the significant correlations highlighted between certain behavioral risk factors and the severity of metabolic syndrome as well as pulmonary dysfunctions. Thus, increased consumption of hyper-caloric ultra-processed foods, reduced consumption of vegetable products, smoking, and a sedentary lifestyle are the main factors that can aggravate obstructive pulmonary diseases associated with metabolic syndrome. Also, the severity of the metabolic syndrome, especially the increase in excess weight and serum lipids, significantly affects lung function.

## 5. Conclusions

Obstructive pulmonary disease is a condition that can endanger the patient’s life if risk factors with an aggravating effect are not effectively monitored. The clinical study carried out on patients with pulmonary function impairment highlighted that metabolic syndrome is an aggravating factor, which, as it becomes more intense, progressively affects pulmonary function in a negative sense. Behavioral risk factors can also play an important role in the deterioration of lung function; smoking, alcohol consumption, and consumption of ultra-processed and hyper-caloric foods increase the risk of metabolic syndrome and worsening respiratory dysfunction.

An unhealthy lifestyle and an unbalanced diet produce, in the long term, a series of imbalances in the body that worsen over time and trigger a series of pathologies with a risk of premature death. One of the most frequent consequences is represented by metabolic syndrome, which, as it worsens, generates a series of associated pathologies that significantly affect the quality of life and even its duration.

## Figures and Tables

**Table 1:** Demographic, anthropometric characteristics, and behavioral factors in correlation with the risk of metabolic syndrome and obstructive pulmonary disease.

Variables		Metabolic Syndrome				
		Yes N = 48		No N = 22		
		Count	Column N %	Count	Column N %	Chi-Square Test Values
Gender	Female	19	39.6%	5	22.7%	χ^2^ = 3.91, *p* = 0.168
	Male	29	60.4%	17	77.3%	
Smoke	Yes	11	22.9%	11	50.0%	χ^2^ = 5.185, *p* = 0.075
	Former	22	45.8%	7	31.8%	
	No	15	31.2%	4	18.2%	
BMI	Normal	9	18.8%	14	63.6%	χ^2^ = 15.85, *p* = 0.001
	Obesity	21	43.8%	2	9.1%	
	Overweight	17	35.4%	5	22.7%	
	Underweight	1	2.1%	1	4.5%	
Sport	Yes, 2–3 times a week	3	6.2%	0	0.0%	χ^2^ = 2.12, *p* = 0.713
	Yes, very rarely	3	6.2%	2	9.1%	
	Yes, daily at least one hour	5	10.4%	2	9.1%	
	Yes, daily for under an hour	1	2.1%	0	0.0%	
	No	36	75.0%	18	81.8%	
Work	Other	2	4.2%	0	0.0%	χ^2^ = 6.266, *p* = 0.394
	Work with extended hours or night shifts	3	6.2%	0	0.0%	
	Work outdoors under normal conditions	8	16.7%	1	4.5%	
	Work in difficult and dangerous conditions (construction site, factory, mine, etc.)	22	45.8%	13	59.1%	
	Work in front of the computer or special devices	4	8.3%	1	4.5%	
	Office work or minimal activity	5	10.4%	3	13.6%	
	Work mostly standing	4	8.3%	4	18.2%	
Eating habits	I believe that I eat chaotically, insufficiently	3	6.2%	5	22.7%	χ^2^ = 4.76, *p* = 0.092
	I consider that I eat chaotically, in excess	2	4.2%	0	0.0%	
	Weighted food consumption, without excesses	43	89.6%	17	77.3%	
Spirometry	DVM	29	60.41%	15	68.18%	χ^2^ = 75.28, *p* < 0.001
	DVO	4	8.33%	5	22.72%	
	DVR	10	20.83%	0	0.00%	
	Normal	5	10.41%	2	9.09%	

Legend: DVM = mixed respiratory dysfunction; DVO = obstructive respiratory dysfunction; DVR = restrictive respiratory dysfunction; Normal = normal pattern on spirometry.

**Table 2:** Cigarette consumption in smokers and the percentage of predicted values for forced expiratory volume in one second (FEV1).

Metabolic Syndrome Risk Score	Age	BMI (kg/m²)	FEV1%	Number of Packs-Years
	Mean ± SD			
0	55.50 ± 10.6	20.70 ± 0.56	67.00 ± 2.82	20.00 ± 0.00
1	57.00 ± 11.93	22.94 ± 2.86	36.00 ± 20.33	33.00 ± 0.00
2	57.26 ± 14.41	24.43 ± 4.37	45.13 ± 24.58	23.00 ± 12.53
3	63.25 ± 9.14	25.67 ± 7.33	55.25 ± 25.77	13.00 ± 4.76
4	63.25 ± 9.83	29.06 ± 5.31	48.66 ± 22.97	17.00 ± 0.00
5	66.54 ± 8.37	31.56 ± 6.16	35.25 ± 20.39	29.66 ± 9.30

**Table 3:** Correlation matrix (Spearman’s rank correlation coefficient) between the parameters that influence the risk of metabolic syndrome.

Variables	Metabolic Syndrome	Age	Fasting Blood Glucose	Abdominal Circumference	BMI kg/m²	TGL	HDL-Cholesterol	FEV1 or VEMS	TA S (mmHg)	TA D (mmHg)
Metabolic syndrome	**1**	**0.304**	**0.394**	**0.502**	**0.431**	**0.498**	**−0.361**	0.199	0.083	0.050
Age	**0.304**	**1**	0.089	0.089	−0.116	0.136	−0.158	−0.113	0.214	−0.002
Fasting blood glucose	**0.394**	0.089	**1**	**0.270**	0.230	**0.318**	−0.189	0.081	−0.225	0.0708
Abdominal circumference	**0.502**	0.089	**0.270**	**1**	**0.891**	**0.316**	**−0.269**	0.110	0.162	0.033
BMI (kg/m²)	**0.431**	−0.115	0.230	**0.891**	**1**	**0.321**	−0.200	**0.310**	0.103	0.042
TGL	**0.498**	0.136	**0.318**	**0.316**	**0.321**	**1**	−0.230	0.219	0.163	0.023
HDL-cholesterol	**−0.361**	−0.156	−0.189	**−0.269**	−0.200	−0.230	**1**	−0.012	−0.161	−0.056
FEV1 or VEMS	0.199	−0.113	0.081	0.110	**0.310**	0.219	−0.012	**1**	**−0.235**	−0.183
TA S (mmHg)	0.083	0.214	−0.225	0.162	0.103	0.163	−0.161	**−0.235**	**1**	**0.479**
TA D (mmHg)	0.050	−0.002	0.070	0.033	0.042	0.023	−0.056	−0.183	**0.479**	**1**

Values in bold are different from 0 with a significance level alpha = 0.05; TA S = systolic blood pressure; TA D = diastolic blood pressure.

## Data Availability

Data are contained within the article.
